# Supplementary material for: Genetic coupling of enhancer activity and connectivity in gene expression control
Source: Nat Commun. 2025 Jan 27;16:970. doi: 10.1038/s41467-025-55900-3 (PMC11772589; doi:10.1038/s41467-025-55900-3)
Supplement: Supplementary file 1 — Supplementary Information [file 41467_2025_55900_MOESM1_ESM.pdf]

Ray-Jones et al., Genetic Coupling of Enhancer Activity and Connectivity in Gene Expression Control.

Supplementary Figures 1-7

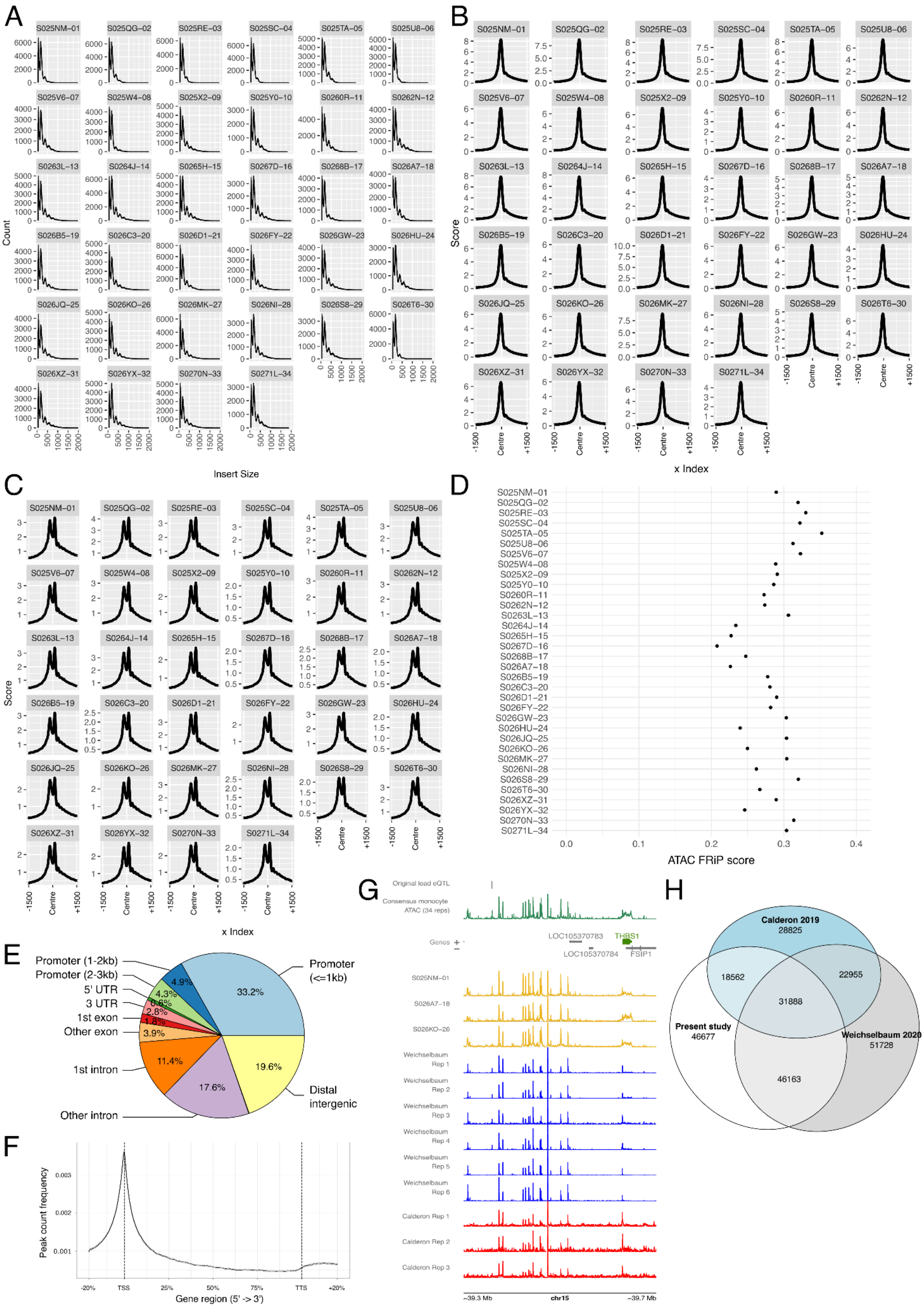

A
B
C
D
E
F
G
H
S025NM-01
S025QG-02
S025RE-03
S025SC-04
S025TA-05
S025U8-06
S025V6-07
S025W4-08
S025X2-09
S025Y0-10
S0260R-11
S0262N-12
S0263L-13
S0264J-14
S0265H-15
S0267D-16
S0268B-17
S026A7-18
S026B5-19
S026C3-20
S026D1-21
S026FY-22
S026GW-23
S026HU-24
S026JQ-25
S026KO-26
S026MK-27
S026NI-28
S026S8-29
S026T6-30
S026XZ-31
S026YX-32
S0270N-33
S0271L-34
Count
Insert Size
Score
x Index
-1500
Centre
+1500
ATAC FRiP score
Promoter (1-2kb)
Promoter (2-3kb)
5' UTR
3 UTR
1st exon
Other exon
1st intron
Other intron
Promoter (<=1kb)
Distal intergenic
33.2%
19.6%
17.6%
11.4%
3.9%
1.8%
2.8%
0.6%
4.3%
4.9%
Peak count frequency
Gene region (5' -> 3')
TSS
TTS
Original lead eQTL
Consensus monocyte ATAC (34 reps)
Genes
LOC105370783
LOC105370784
THBS1
FSIP1
Weichselbaum Rep 1
Weichselbaum Rep 2
Weichselbaum Rep 3
Weichselbaum Rep 4
Weichselbaum Rep 5
Weichselbaum Rep 6
Calderon Rep 1
Calderon Rep 2
Calderon Rep 3
~39.3 Mb
chr15
~39.7 Mb
Calderon 2019
28825
Present study
46677
Weichselbaum 2020
51728
18562
22955
31888
46163

Supplementary Figure 1. Quality control of ATAC-seq on fixed chromatin in primary monocytes. *A) Fragment size plots of ATAC-seq libraries for each replicate. B) Signal profiles of nucleosome-free ATAC-seq reads at transcription start sites for each replicate. C) Signal profiles of mononucleosome ATAC-seq reads at transcription start sites for each replicate. D) Fraction of read in peak (FRiP) score for each ATAC-seq replicate. E) Intersection of ATAC-seq peaks with genomic region annotations (HMMRATAC peaks called using consensus dataset of all 34 replicates). F) Average profile of consensus ATAC peaks binding to genic regions, from 20% upstream of the transcription start site (TSS) to 20% downstream of the transcription termination site (TTS). The 95% confidence interval is shown as a grey ribbon. G) Comparison of ATAC-seq signal in the THBS1 locus: consensus pileup (all 34 replicates), three primary monocyte replicates from the present study and individual replicate-level signal from publicly available primary monocyte ATAC-seq data (Weichselbaum et al., 2020; Calderon et al., 2019). H) Intersecting ATAC-seq peaks between consensus datasets for the present study, Weichselbaum et al. and Calderon et al., determined using Bedtools multiinter on HMRRATAC peaks for all three studies. FRiP, fraction of reads in peak; TSS, transcription start site; TTS, transcription termination site. Source data are for panels D, E, F and H are available on OSF[167].*

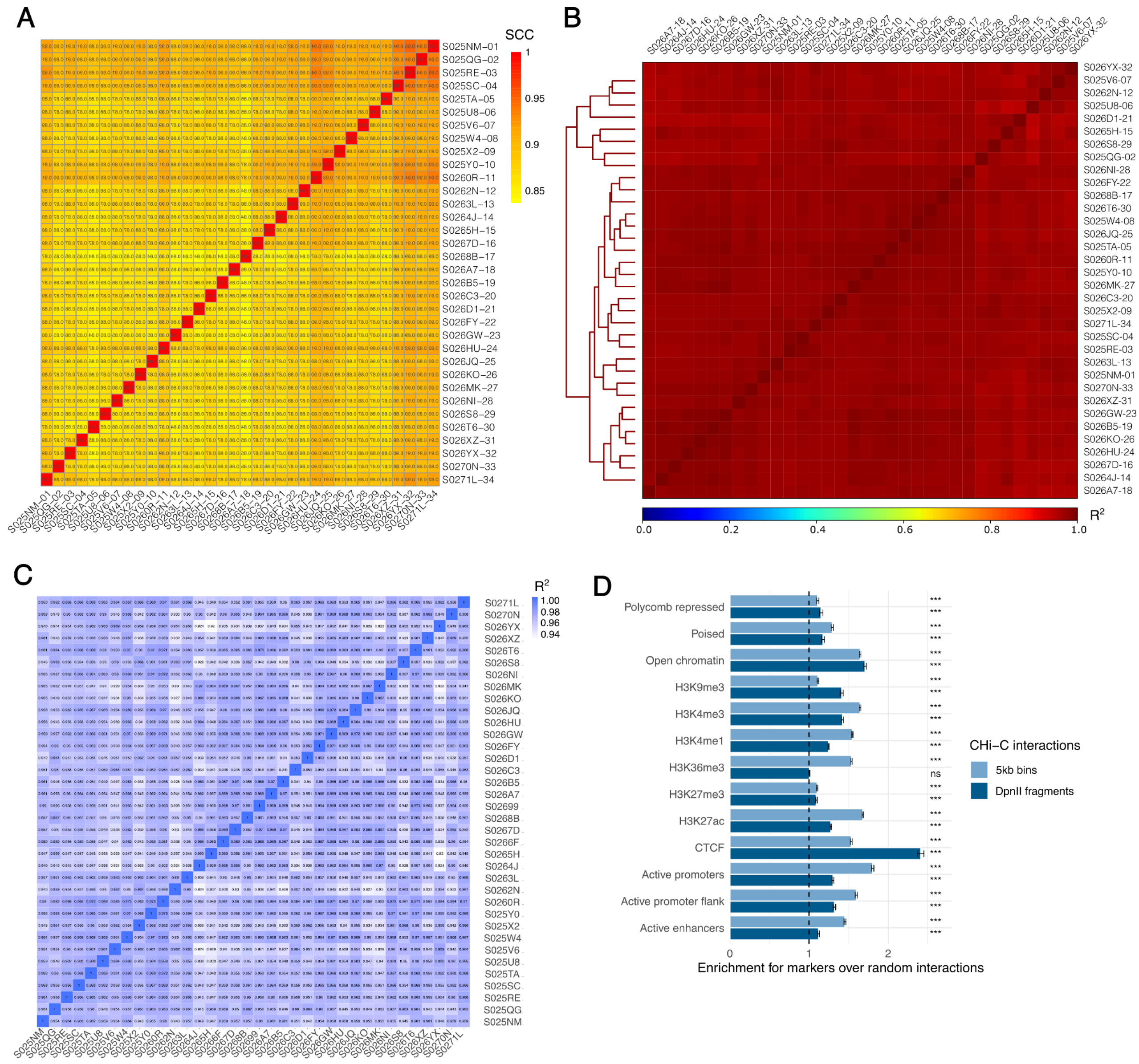

Supplementary Figure 2. QC of CHi-C, ATAC-seq and RNA-seq samples. *A) HiCRep results showing stratum-adjusted correlation coefficient (SCC) values for CHi-C data on chromosome 22. B) Pearson's correlation values for ATAC-seq samples (deepTools). C) Pearson's correlation between RNA samples. Note that sample S02699 was a technical replicate of S026T6. In addition, sample S0266F was a sample that failed genotyping due to a very low call rate, so it was not included in any further analyses. D) CHi-C enrichments at other ends of significant interactions at the resolution of 5kb binned regions (light blue) or DpnII fragments (dark blue). The enrichment was determined for each marker against randomly sampled chromatin fragments located the same distance away from the baited regions as the detected interacting fragments, using the peakEnrichment4Features function in Chicago[33,34]. Three asterisks (***) denote one-sided p-values below 0.001 and "ns" represents "non-significant" (permutation test with 100 random samples). Error bars show the standard deviation of the mean ratio between true interacting*

fragments and the random interacting fragments. Histone modification peaks were obtained from Blueprint. Further annotations (promoter flanking regions, promoters, enhancers, poised and polycomb repressed regions) were taken from Blueprint projected monocyte segmentations. ATAC-seq peaks of open chromatin were obtained from the present monocyte cohort (consensus HMMRATAC peaks). Source data for all panels are available on OSF[167].

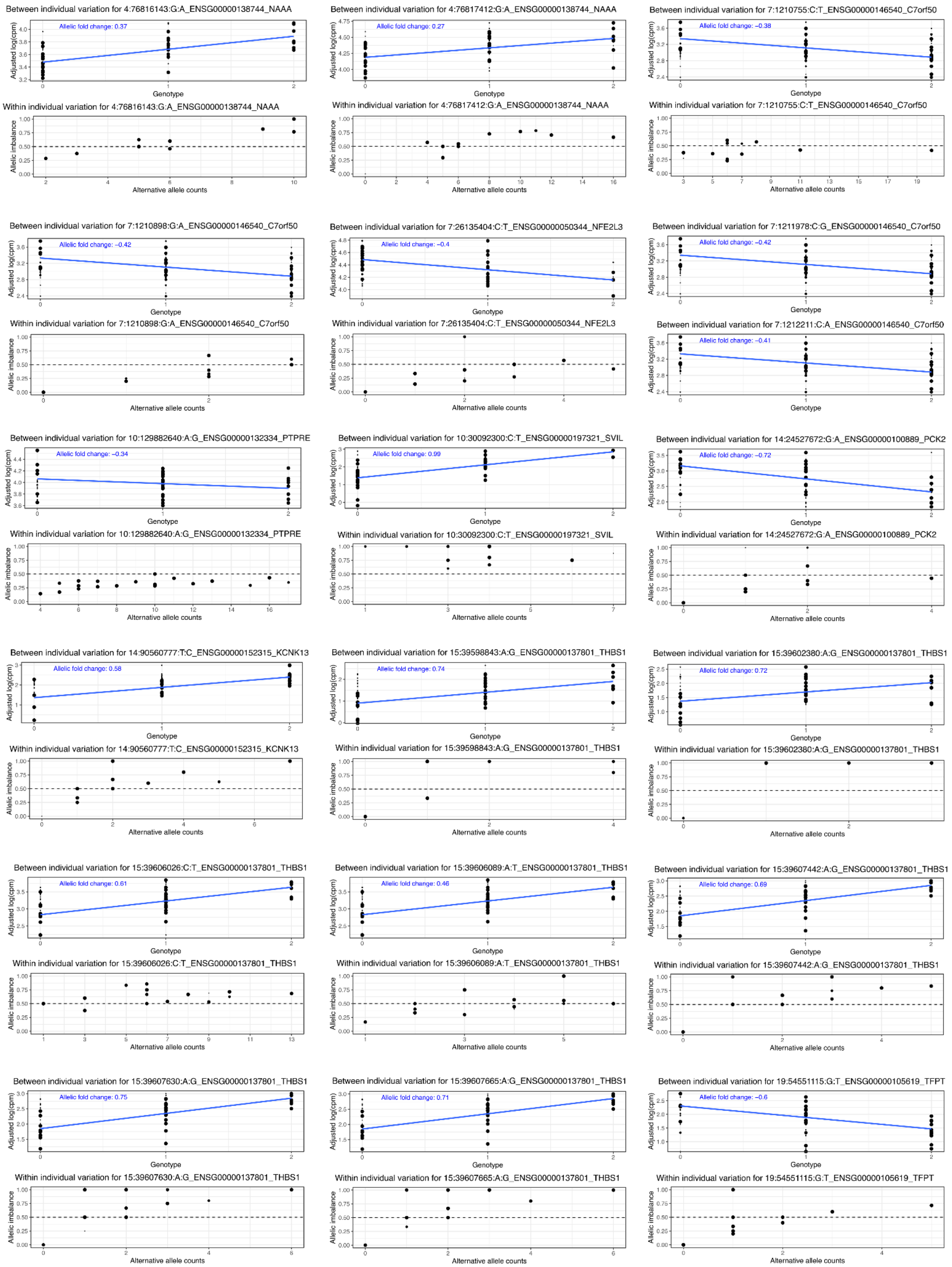
Between individual variation for 4:76816143:G:A_ENSG00000138744_NAAA
Allelic fold change: 0.37
Within individual variation for 4:76816143:G:A_ENSG00000138744_NAAA
Between individual variation for 4:76817412:G:A_ENSG00000138744_NAAA
Allelic fold change: 0.27
Within individual variation for 4:76817412:G:A_ENSG00000138744_NAAA
Between individual variation for 7:1210755:C:T_ENSG00000146540_C7orf50
Allelic fold change: −0.38
Within individual variation for 7:1210755:C:T_ENSG00000146540_C7orf50
Between individual variation for 7:1210898:G:A_ENSG00000146540_C7orf50
Allelic fold change: −0.42
Within individual variation for 7:1210898:G:A_ENSG00000146540_C7orf50
Between individual variation for 7:26135404:C:T_ENSG00000050344_NFE2L3
Allelic fold change: −0.4
Within individual variation for 7:26135404:C:T_ENSG00000050344_NFE2L3
Between individual variation for 7:1211978:C:G_ENSG00000146540_C7orf50
Allelic fold change: −0.42
Between individual variation for 7:1212211:C:A_ENSG00000146540_C7orf50
Allelic fold change: −0.41
Between individual variation for 10:129882640:A:G_ENSG00000132334_PTPRE
Allelic fold change: −0.34
Within individual variation for 10:129882640:A:G_ENSG00000132334_PTPRE
Between individual variation for 10:30092300:C:T_ENSG00000197321_SVIL
Allelic fold change: 0.99
Within individual variation for 10:30092300:C:T_ENSG00000197321_SVIL
Between individual variation for 14:24527672:G:A_ENSG00000100889_PCK2
Allelic fold change: −0.72
Within individual variation for 14:24527672:G:A_ENSG00000100889_PCK2
Between individual variation for 14:90560777:T:C_ENSG00000152315_KCNK13
Allelic fold change: 0.58
Within individual variation for 14:90560777:T:C_ENSG00000152315_KCNK13
Between individual variation for 15:39598843:A:G_ENSG00000137801_THBS1
Allelic fold change: 0.74
Within individual variation for 15:39598843:A:G_ENSG00000137801_THBS1
Between individual variation for 15:39602380:A:G_ENSG00000137801_THBS1
Allelic fold change: 0.72
Within individual variation for 15:39602380:A:G_ENSG00000137801_THBS1
Between individual variation for 15:39606026:C:T_ENSG00000137801_THBS1
Allelic fold change: 0.61
Within individual variation for 15:39606026:C:T_ENSG00000137801_THBS1
Between individual variation for 15:39606089:A:T_ENSG00000137801_THBS1
Allelic fold change: 0.46
Within individual variation for 15:39606089:A:T_ENSG00000137801_THBS1
Between individual variation for 15:39607442:A:G_ENSG00000137801_THBS1
Allelic fold change: 0.69
Within individual variation for 15:39607442:A:G_ENSG00000137801_THBS1
Between individual variation for 15:39607630:A:G_ENSG00000137801_THBS1
Allelic fold change: 0.75
Within individual variation for 15:39607630:A:G_ENSG00000137801_THBS1
Between individual variation for 15:39607665:A:G_ENSG00000137801_THBS1
Allelic fold change: 0.71
Within individual variation for 15:39607665:A:G_ENSG00000137801_THBS1
Between individual variation for 19:54551115:G:T_ENSG00000105619_TFPT
Allelic fold change: −0.6
Within individual variation for 19:54551115:G:T_ENSG00000105619_TFPT
Adjusted log(cpm)
Genotype
Allelic imbalance
Alternative allele counts

Supplementary Figure 3. BaseQTL estimated effects for CHi-C QTLs. *For each of the 19 significant BaseQTL contact eQTLs, we illustrate the two components of our model: between-individual (top) and within-individual (bottom) variation. Between-individual plots show the genotype of the cis-SNP (x-axis) against the total counts adjusted by library size in log scale (y-axis). The size of each point corresponds to the probability of the indicated genotype. Only heterozygotes are considered to represent the within-individual variation. The x-axis corresponds to the number of reads mapping the cis-SNP alternative allele, while the y-axis shows the proportion of reads mapping the cis-SNP alternative allele. The size of each point corresponds to the probability of the cis-SNP genotype being heterozygous. The value of the estimated log allelic fold change is given in blue. Only between-individual information was available to model the associations between cis-SNPs 7:1211978:C:G and 7:1212211:C:A with the promoter of C7orf50. Source data for this figure cannot be released due to donor consent conditions as it contains raw genotype information.*

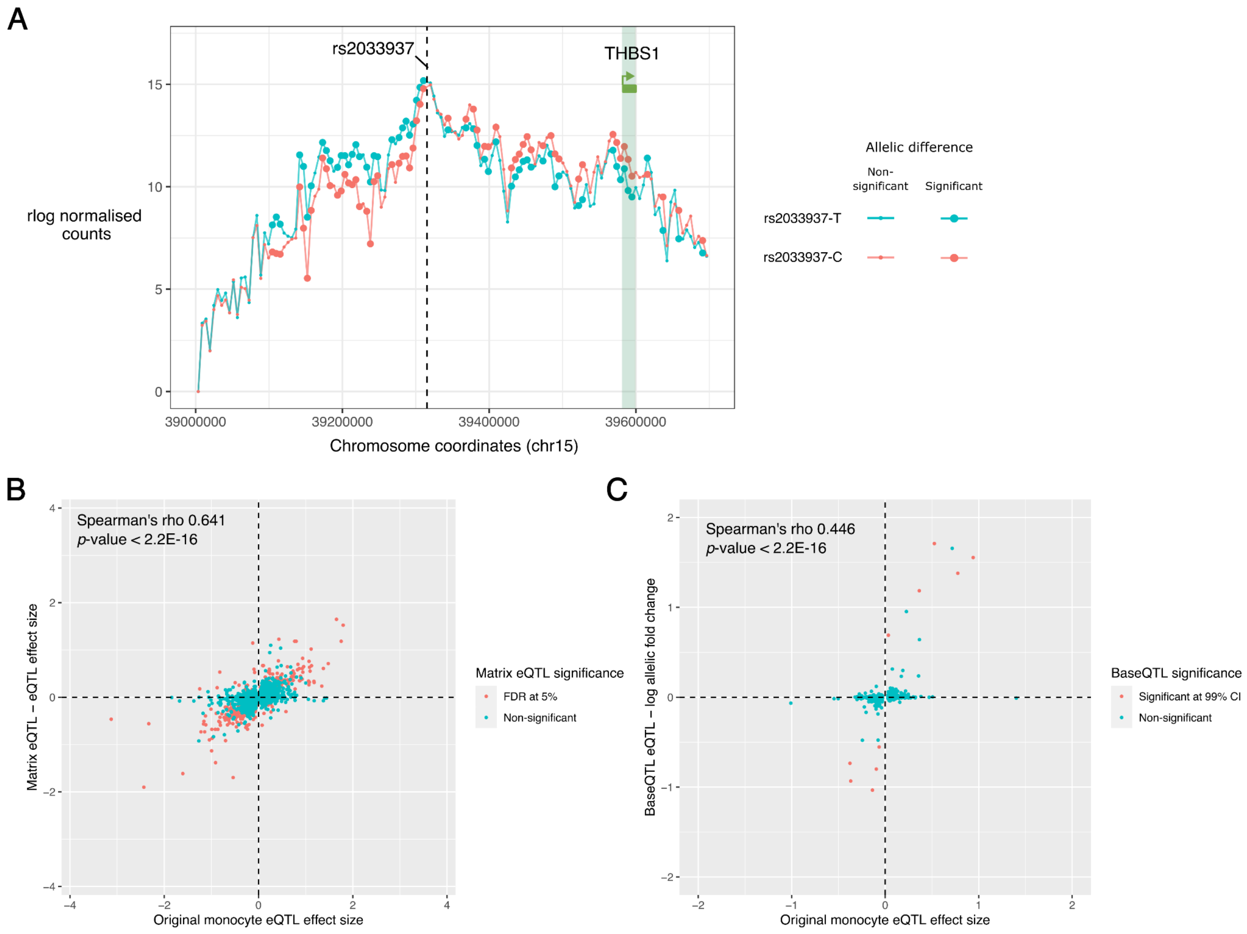

Supplementary Figure 4. Validation of a chromatin QTL at THBS1 and correlation between eQTL effects in the original multi-cohort monocyte study and in the present cohort*. A) 4C-seq validation of allele-specific chromatin conformation between the eQTL rs2033937 and its eGene THBS1. The mean 4C-seq signal across three heterozygous individuals (rlog normalised reads) is shown for the reference allele (T, blue) and the alternative allele (C, red) of rs2033937. Significantly different interactions are shown as large dots (4Cker adjusted p-value < 0.05 after adjusting for multiple testing). B) Correlation of the original eQTL dataset with eQTLs called by Matrix eQTL in the present study. C) Correlation of the original eQTL dataset with eQTLs called by BaseQTL in the present study. Source data for all panels are available on OSF[167].*

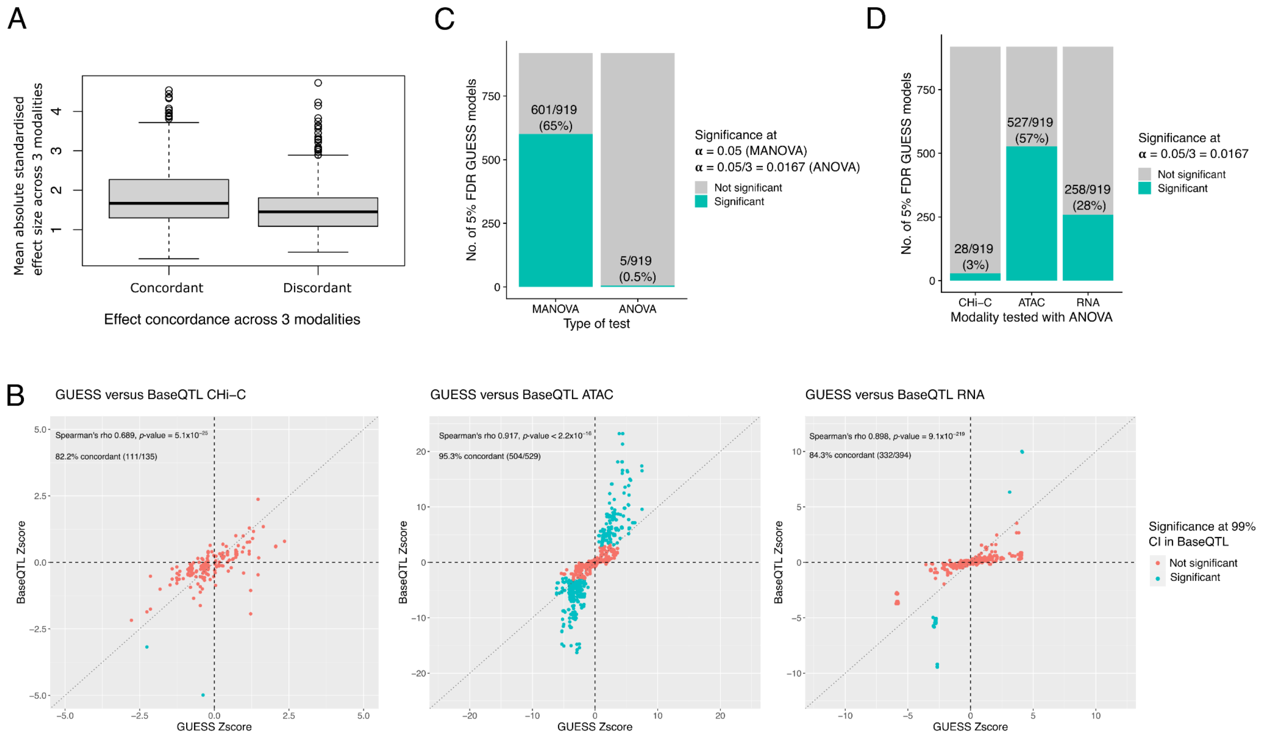

Supplementary Figure 5. Validation of GUESS results. *A). Standardised effect size of GUESS models depending on overall concordance. In concordant models, the sign of the beta was the same for all three modalities (CHi-C, ATAC-seq and RNA-seq). For discordant models, at least one of the signs was different. Only GUESS models containing one trimodal QTL at 5% FDR were included in this analysis. Boxplots show 25th, 50th and 75th percentiles, with upper and lower whiskers to the largest or smallest value no further than 1.5 x the interquartile range from the hinge. B) GUESS and BaseQTL concordance. The GUESS data (FDR 5%, single QTL models) and BaseQTL data (all results) were merged on the trimodal QTL and the feature: CHi-C bait fragment (left), ATAC-seq peak (middle) or gene (right). The results are coloured by their significance in BaseQTL at a CI cutoff of 0.99. Only single-QTL models in GUESS were used for the comparisons in this figure. Concordance is defined as a QTL having the same direction of effect across analyses. C) We performed multivariate analysis of variance (MANOVA) on the lead trimodal QTL identified by GUESS in each window, treating the other trimodal QTLs in the same window (if detected at 5% FDR) as covariates (MANCOVA), with the graph showing the number of models at alpha = 0.05. We also assessed how many of the lead trimodal QTLs could be validated using a univariate approach (ANOVA/ANCOVA), with the graph showing the number of models at alpha = 0.0167 for all three modalities (adjusting for multiple testing). D) Validation of each of the three individual modalities using ANOVA/ANCOVA (alpha = 0.0167). Source data for all panels are available on OSF[167].*

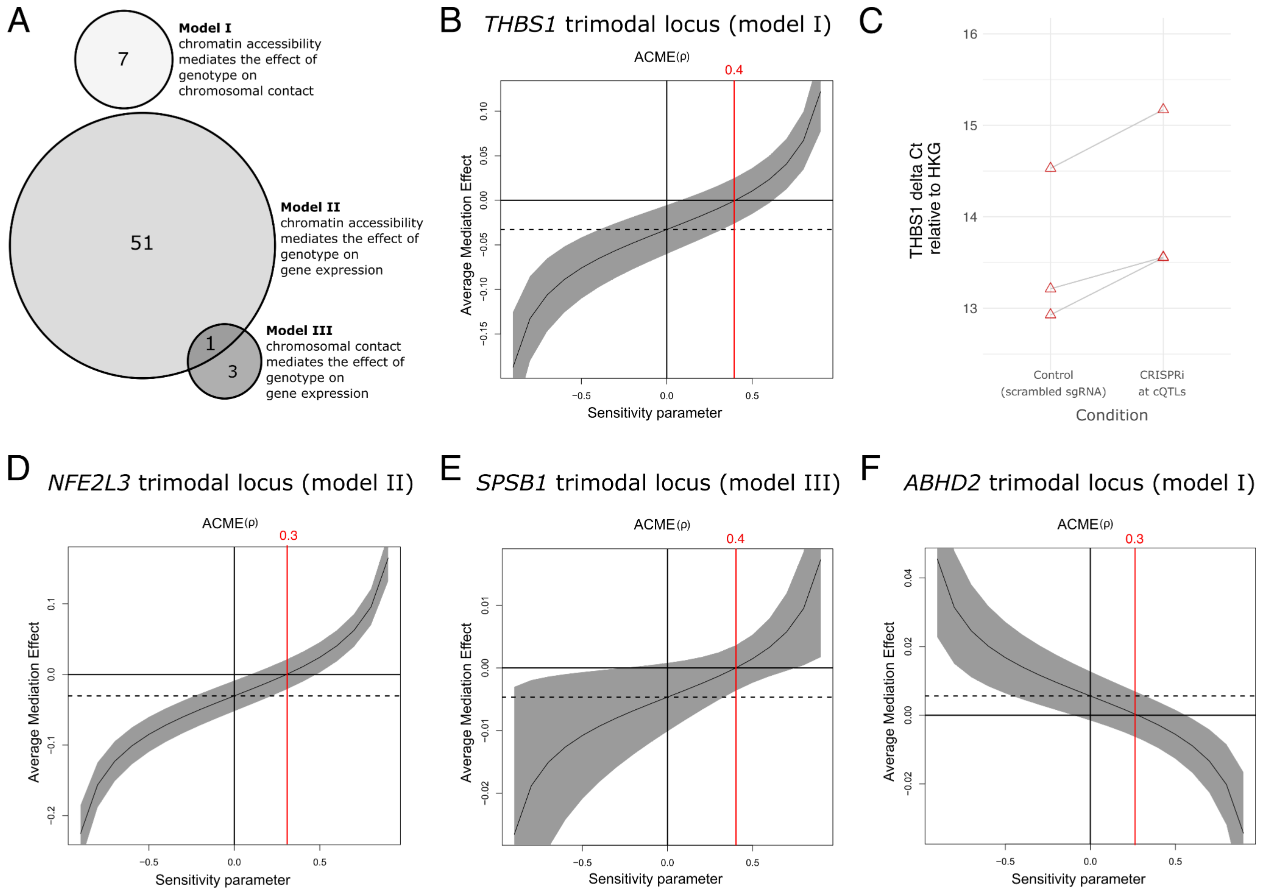

Supplementary Figure 6. Mediation models. *A) Total Number of trimodal QTLs implicated in each mediation model (full results in Supplementary Data 11). B). Sensitivity plot testing for the existence of unobserved post-treatment confounders in the THBS1 locus. The plot shows the values of causal quantities as a function of sensitivity parameter (the correlation between the residuals of the mediator and outcome regressions). The solid line in the curve represents the estimated average mediation effect at different sensitivity parameter values, with the grey areas representing the 95% confidence interval. The ACME estimate is shown as a black horizontal dotted line. The red vertical line shows the level of confounding required to observe no mediation effect (ACME = 0) and, thus, invalidate the results. Values ≥ 0.3 indicate that a strong confounding effect is necessary to change the sign of ACME estimates. C) Delta Ct values for the THSB1 expression qPCR in the CRISPRi experiment, compared with the housekeeping genes (HKG) GAPDH, TOP1 and ATP. D-F) same as B but for the NFE2L3, SPSB1 and ABDH2 loci. Source data for panel C are provided in the Source Data spreadsheet. Source data for panels B, D-F could not be obtained as they were generated automatically by the R package mediation as part of the analysis shown in Figs. 5E, J, L and 7C.*

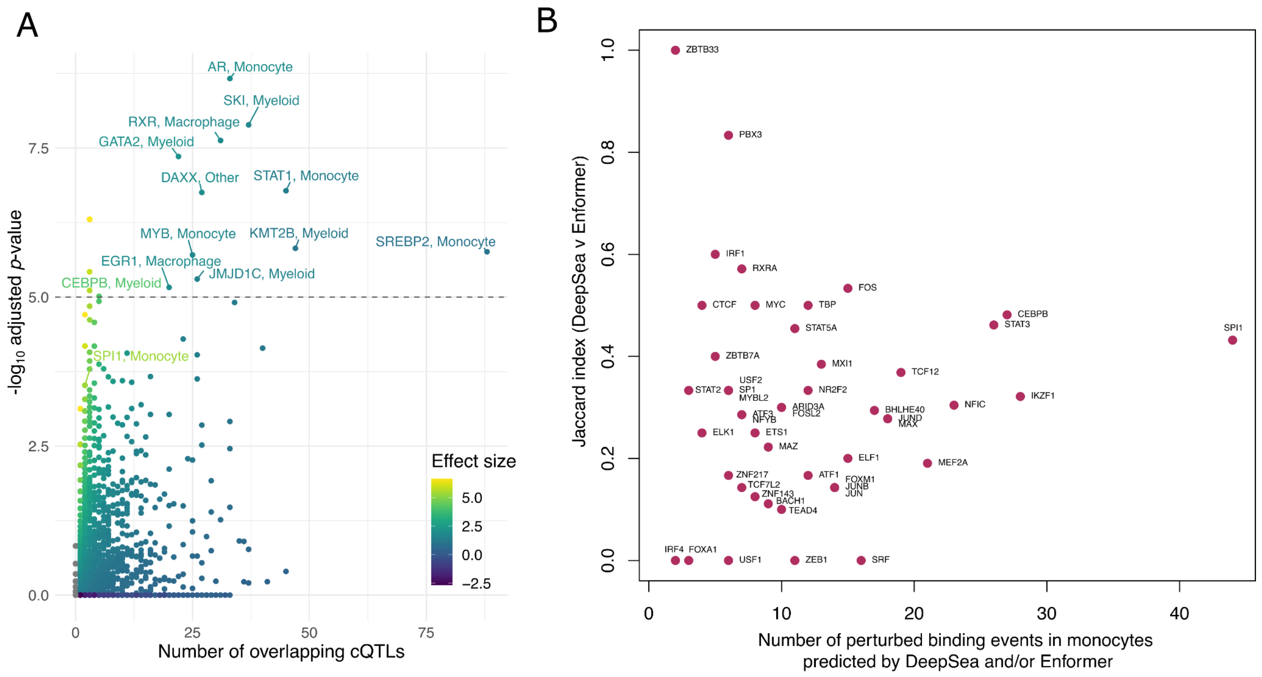

Supplementary Figure 7. cQTLs intersect and are predicted to perturb TF binding sites*. A) Enrichment of experimentally determined TF binding sites at cQTLs (ReMap 2022 ChIP-seq dataset). TFs are labelled at -log10 adjusted p-value (Q significance) > 5 and number of overlapping cQTLs >= 5, apart from SPI1, which is shown for reference, because it was also identified in the analysis of monocyte-specific ChIP-seq and ATAC-seq predicted peaks. The statistical tests were performed using Remapenrich. Each cell type was classified as "monocyte", "macrophage", "myeloid" or "other". B) Jaccard index of DeepSea vs Enformer is shown against the number of predicted perturbed binding events in monocytes at cQTLs, as predicted by DeepSea and/or Enformer. Source data for both panels are available on OSF[167].*
