# Supplementary material for: Genetic coupling of enhancer activity and connectivity in gene expression control
Source: Nat Commun. 2025 Jan 27;16:970. doi: 10.1038/s41467-025-55900-3 (PMC11772589; doi:10.1038/s41467-025-55900-3)
Supplement: Supplementary file 3 — Description of Additional Supplementary Files [file 41467_2025_55900_MOESM3_ESM.pdf]

Ray-Jones et al., Genetic Coupling of Enhancer Activity and Connectivity in Gene Expression Control

Description of Supplementary Data

Supplementary Data 1. Monocyte meta-analysis eQTLs used for eQTL Capture Hi-C design.
Supplementary Data 2. eQTL CHi-C QC summary statistics
Supplementary Data 3. ATAC-seq QC summary statistics
Supplementary Data 4. RNA-seq QC summary statistics
Supplementary Data 5. The locations of TADs in primary monocytes detected from Hi-C data
Supplementary Data 6. CHi-C ABC analysis results
Supplementary Data 7. BaseQTL results for CHi-C data (Tab A: Significant contact eQTL; Tab B: Contact eQTL summary statistics; Tab C: Contact non eQTL summary statistics)
Supplementary Data 8. BaseQTL results for ATAC-seq data
Supplementary Data 9. BaseQTL results for RNA-seq data
Supplementary Data 10. Trimodal QTL detection by GUESS analysis results
Supplementary Data 11. Causal mediation analysis results
Supplementary Data 12. TF enrichment analysis results (Tab A: Enrichment for known and predicted binding sites of monocyte TFs; Tab B: Enrichment for known binding sites of all TFs contained in the Remap database)
Supplementary Data 13. Prediction of cQTL effects on TF binding (Tab A: consensus predictions; Tab B: Enformer predictions; Tab C: DeepSea predictions, Tab D: Known and predicted TF binding sites overlapping cQTLs)
Supplementary Data 14. cQTL integration with GWAS SNPs (Tab A: Intersection of cQTLs with GWAS SNPs accounting for LD; Tab B: Additional information on white blood cell GWAS SNPs overlapping cQTLs).
Supplementary Data 15. Monocyte sample information
