# Supplementary material for: Genetic coupling of enhancer activity and connectivity in gene expression control
Source: Nat Commun. 2025 Jan 27;16:970. doi: 10.1038/s41467-025-55900-3 (PMC11772589; doi:10.1038/s41467-025-55900-3)
Supplement: Supplementary file 19 — Reporting Summary [file 41467_2025_55900_MOESM19_ESM.pdf]

nature portfolio

Corresponding author(s): Mikhail Spivakov, Helen Ray-Jones

Last updated by author(s): 11/25/2024

Reporting Summary

Nature Portfolio wishes to improve the reproducibility of the work that we publish. This form provides structure for consistency and transparency in reporting. For further information on Nature Portfolio policies, see our Editorial Policies and the Editorial Policy Checklist.

Statistics

For all statistical analyses, confirm that the following items are present in the figure legend, table legend, main text, or Methods section.

n/a	Confirmed	
☐	☒	The exact sample size (*n*) for each experimental group/condition, given as a discrete number and unit of measurement
☐	☒	A statement on whether measurements were taken from distinct samples or whether the same sample was measured repeatedly
☐	☒	The statistical test(s) used AND whether they are one- or two-sided<br>*Only common tests should be described solely by name; describe more complex techniques in the Methods section.*
☐	☒	A description of all covariates tested
☐	☒	A description of any assumptions or corrections, such as tests of normality and adjustment for multiple comparisons
☐	☒	A full description of the statistical parameters including central tendency (e.g. means) or other basic estimates (e.g. regression coefficient) AND variation (e.g. standard deviation) or associated estimates of uncertainty (e.g. confidence intervals)
☐	☒	For null hypothesis testing, the test statistic (e.g. *F*, *t*, *r*) with confidence intervals, effect sizes, degrees of freedom and *P* value noted<br>*Give P values as exact values whenever suitable.*
☐	☒	For Bayesian analysis, information on the choice of priors and Markov chain Monte Carlo settings
☒	☐	For hierarchical and complex designs, identification of the appropriate level for tests and full reporting of outcomes
☐	☒	Estimates of effect sizes (e.g. Cohen's *d*, Pearson's *r*), indicating how they were calculated

Our web collection on statistics for biologists contains articles on many of the points above.

Software and code

Policy information about availability of computer code

Data collection: No software was used for data collection.

Data analysis: Custom scripts are available at: https://github.com/FunctionalGeneControl/contactQTLs (eQTL CHi-C design, data processing and R scripts for running GUESS [analysis, post-processing and MANCOVA/ANCOVA comparisons]), https://github.com/pavarte/PCHIC-ABC-Prediction (CHi-C ABC analysis), https://gitlab.com/evigorito/baseqtl_atac_chic_eqtl (BaseQTL extended to CHi-C and ATAC-seq modalities). A stable release of the analysis code for this paper is available at DOI: 10.5281/zenodo.14210247.

R libraries (available from CRAN or Bioconductor unless otherwise specified): Chicago v1.28.0, BaseQTL (https://gitlab.com/evigorito/baseqtl), R2GUESS (https://github.com/lb664/R2GUESS), pROC v1.18.4 , glmnet v4.1-8, motifbreakR v2.14.2, cluster v2.14, ReMapEnrich v0.99.0 (https://remap-cisreg.github.io/ReMapEnrich/), DESeq2 v1.40.2, MatrixEQTL v2.3, Rsubread v2.14.2, soGGi v1.18.0, CEMiTool v0.3, HiCRep v1.10.0, PlotGardener v1.2.10 (https://phanstiellab.github.io/plotgardener/), motifmatchr v1.22.0, motifDB v1.42.0, randomForest v4.7-1.1, R.4Cker v1.0 (https://github.com/rr1859/R.4Cker), ChIPSeeker v1.42.0, mediation v4.5.0.

Python libraries: DeepTools v3.3.1 (obtained from bioconda), kipoiseq v0.5.2, tensorflow v2.9.2, tensorflow-hub v0.12.0. The pre-trained Enformer model “TF2.0 Saved Model (v1)” was downloaded on 26th January 2023 from https://tfhub.dev/deepmind/enformer/1. The python tool bamsplit was obtained from https://github.com/luntergroup/bamsplit.

Software: R v4; python v2.7, v3.7; plink v1.07 (https://www.cog-genomics.org/plink/), Hi-C User Pipeline v0.7.4 (https://github.com/StevenWingett/HiCUP/tree/combinations), Homer Toolkit v4.10.4 (http://homer.ucsd.edu/homer/download.html), TOBIAS (https://github.com/loosolab/TOBIAS_snakemake; downloaded April 2023), phASER v1.1.1 (https://github.com/secastel/phaser), BEDtools v2.30.0, SAMtools v1.3.1, bcftools v1.3.1, Michigan Imputation Server v1.2.4 (https://imputationserver.sph.umich.edu/), HMMRATAC v1.2.10 (https://

github.com/LiuLabUB/HMMRATAC), STAR v2.6.1d (https://github.com/alexdobin/STAR), Picard v2.6.0, Bowtie2 v2.2.9, NGmerge v0.3 obtained from bioconda), pipe4C v1.1.6 (https://github.com/deLaatLab/pipe4C), SeqKit (https://github.com/shenwei356/seqkit), ChIP-AP v5.4 (https://github.com/JSuryatenggara/ChIP-AP), maxATAC v1.0.5 (https://github.com/MiraldiLab/maxATAC/tree/main), DeepSea predictions were queried using the online interface (https://hb.flatironinstitute.org/deepsea/).

For manuscripts utilizing custom algorithms or software that are central to the research but not yet described in published literature, software must be made available to editors and reviewers. We strongly encourage code deposition in a community repository (e.g. GitHub). See the Nature Portfolio guidelines for submitting code & software for further information.

Data

Policy information about availability of data

All manuscripts must include a data availability statement. This statement should provide the following information, where applicable:

- Accession codes, unique identifiers, or web links for publicly available datasets
- A description of any restrictions on data availability
- For clinical datasets or third party data, please ensure that the statement adheres to our policy

4C-seq and ATAC-seq data from U-937 cells are deposited in GEO for unrestricted access (accession numbers GSE281908 [https://www.ncbi.nlm.nih.gov/geo/query/acc.cgi?acc=GSE281908] and GSE281909 [https://www.ncbi.nlm.nih.gov/geo/query/acc.cgi?acc=GSE281909]). Large supplementary data (such as eQTL CHi-C design, CHi-C significant interactions, ATAC-seq peaks and TF footprinting), as well as source data for figure panels are available on the Open Science Framework (OSF) at https://osf.io/szntj/.

Raw sequencing data from human monocytes generated in this study are available in the European Genome-Phenome Archive (EGA) under managed access, in accordance with the donor terms of consent. The data can be found under Dataset ID EGAD50000001116 (https://ega-archive.org/datasets/EGAD50000001116). To request access, please contact the Data Access Committee at eQTL-CHiC-DAC-WC@groups.imperial.ac.uk. An application form will be provided within seven working days of the request. Submitted forms signed by the requestor(s) and an authorised institutional representative will be reviewed within one calendar month, and access will be granted provided all access conditions are met.

Research involving human participants, their data, or biological material

Policy information about studies with human participants or human data. See also policy information about sex, gender (identity/presentation), and sexual orientation and race, ethnicity and racism.

Reporting on sex and gender	34 male platelet donors, age 20 to 70, were recruited to the study between September 2018 and September 2019. Since sex is known to affect monocyte biology, we sought to recruit a single-sex cohort. Only male donors were chosen due to the low number of female platelet donors at the recruitment centre. Sex was self-reported and confirmed by genotyping. Since our cohort was all male, we did not perform sex- or gender-based analyses.
Reporting on race, ethnicity, or other socially relevant groupings	All participants self-identified as white British.
Population characteristics	All study participants were NIHR BioResource volunteers and NHS Blood and Transplant platelet donors.
Recruitment	Participants were recruited after informed consent to obtain the apheresis cone which is normally discarded after platelet donation. The participants were not compensated.
Ethics oversight	All participants signed an informed consent and the study was under the ethical approval "A Blueprint of Blood Cells" (12/EE/0040) approved by the NHS Health Research Authority Research Ethics Committee (REC) East of England-Hertfordshire.

Note that full information on the approval of the study protocol must also be provided in the manuscript.

Field-specific reporting

Please select the one below that is the best fit for your research. If you are not sure, read the appropriate sections before making your selection.

☒ Life sciences ☐ Behavioural & social sciences ☐ Ecological, evolutionary & environmental sciences

For a reference copy of the document with all sections, see nature.com/documents/nr-reporting-summary-flat.pdf

Life sciences study design

All studies must disclose on these points even when the disclosure is negative.

Sample size	Samples from 34 donors were used due to practical feasibility of obtaining material and performing the experiments. No sample size calculation was performed. The sample size of 34, while on the low side compared with population genomics studies, is high in comparison with other Capture Hi-C studies, where the standard sample size is 2-3. To compensate for the sample size, we boosted the statistical power of our data by developing the 'trimodal QTL' approach that leverages correlated molecular phenotypes.

Data exclusions	The initial cohort included 35 samples, but one sample failed genotyping (call rate ~60%) and so only the remaining 34 samples were carried forward in the study.
Replication	We validated the eQTL data used for study design on our cohort (see main text). Two of the contact QTLs identified in the BaseQTL analysis were replicated using an alternative experimental methodology, 4C-seq. We did not test any of the other cQTLs using 4C-seq. The data generated in this study were not replicated on an independent cohort.
Randomization	No randomisation was used. Covariates: For all analyses, read bias for the reference genome was accounted for by filtering reads using the WASP pipeline or by controlling for the bias using RefBias (a part of the BaseQTL workflow). In BaseQTL, total read counts were adjusted by library size by providing it as a covariate. In GUESS, we performed variance stabilisation and normalisation on the total counts for CHi-C, ATAC-seq and RNA-seq using the rlog approach implemented in the R package DESeq2.
Blinding	Blinding was not relevant to the study, because participants were not allocated to groups.

Reporting for specific materials, systems and methods

We require information from authors about some types of materials, experimental systems and methods used in many studies. Here, indicate whether each material, system or method listed is relevant to your study. If you are not sure if a list item applies to your research, read the appropriate section before selecting a response.

Materials & experimental systems

n/a	Involved in the study
☐	☒ Antibodies
☐	☒ Eukaryotic cell lines
☒	☐ Palaeontology and archaeology
☒	☐ Animals and other organisms
☒	☐ Clinical data
☒	☐ Dual use research of concern
☒	☐ Plants

Methods

n/a	Involved in the study
☐	☒ ChIP-seq
☒	☐ Flow cytometry
☒	☐ MRI-based neuroimaging

Antibodies

Antibodies used	Monocyte isolation: STEMCELL Monocyte isolation kit Catalog #19669, anti CD14 FITC, clone MϕP9 , Becton Dickinson, cat 345784; anti CD16 PE, clone B73.1 / leu11c, Becton Dickinson, cat 332779; anti CD64 PerCP-Cy5.5, clone 10.1, Becton Dickinson, cat 561194; anti CD45 PE-CY7, clone HI30, Invitrogen, cat MHCD4512. ChIP seq: CTCF: Merk 07-729
Validation	All antibodies are commercially available and those used for monocyte isolation have been extensively tested including clinical diagnostic use.

Eukaryotic cell lines

Policy information about cell lines and Sex and Gender in Research

Cell line source(s)	HEK 293T cells , used to generate lentivirus, were obtained from other research groups within the MRC LMS and the VIB CMN. The U-937 monocyte-like cell line, which was used for the CRISPRi experiment, was purchased from ECACC (Public Health England) in October 2020. The U-937 cell line comes from a male donor, which matches the male monocyte cohort.
Authentication	None of the cell lines used in this study were authenticated.
Mycoplasma contamination	All cell lines tested negatively for mycoplasma infection
Commonly misidentified lines (See ICLAC register)	None

Plants

Seed stocks	We did not use plants.
Novel plant genotypes	We did not use plants.
Authentication	We did not use plants.

ChIP-seq

Data deposition

☒ Confirm that both raw and final processed data have been deposited in a public database such as GEO.

☒ Confirm that you have deposited or provided access to graph files (e.g. BED files) for the called peaks.

Data access links<br>*May remain private before publication.*	Signal tracks per replicate (.bw files) and CTCF peaks, calculated using ChIP-AP on the three replicates, are available on OSF: doi:10.17605/OSF.IO/SZNTJ Raw sequencing data from human monocytes generated in this study are available in the European Genome-Phenome Archive (EGA) under managed access, in accordance with the donor terms of consent. The data can be found under Dataset ID EGAD50000001116 (https://ega-archive.org/datasets/EGAD50000001116). To request access, please contact the Data Access Committee at eQTL-CHiC-DAC-WC@groups.imperial.ac.uk. An application form will be provided within seven working days of the request. Submitted forms signed by the requestor(s) and an authorised institutional representative will be reviewed within one calendar month, and access will be granted provided all access conditions are met.
Files in database submission	CTCF signal tracks (OSF, doi:10.17605/OSF.IO/SZNTJ):<br>mono_CTCF_chip_rep1.bw<br>mono_CTCF_chip_rep2.bw<br>mono_CTCF_chip_rep3.bw<br>mono_CTCF_ctrl_rep1.bw<br>mono_CTCF_ctrl_rep2.bw<br>mono_CTCF_ctrl_rep3.bw<br><br>CTCF peaks (OSF, doi:10.17605/OSF.IO/SZNTJ): "mono_CTCF_all_peaks_calculated.tsv" - CTCF peaks called from ChIP data from three primary monocyte samples, using the ChIP-AP pipeline.<br><br>Raw sequencing data (EGA, managed access under Dataset ID EGAD50000001116): CTCF ChIP-seq and input chromatin samples for three biological replicates.
Genome browser session<br>(e.g. UCSC)	No longer applicable

Methodology

Replicates	Primary monocytes from three donors
Sequencing depth	150 bp paired end sequencing on a NovaSeq platform (Novogene Europe) was performed for all ChIP-seq samples. Sample information:<br>Rep 1, CTCF: 20,728,818 total reads, 7,070,479 uniquely mapped<br>Rep 2, CTCF: 16,238,503 total reads, 7,855,464 uniquely mapped<br>Rep 3, CTCF: 17,233,720 total reads, 4,033,725 uniquely mapped<br>Rep 1, input: 15,215,878 total reads, 5,657,050 uniquely mapped<br>Rep 2, input: 23,389,847 total reads, 9,445,565 uniquely mapped<br>Rep 3, input: 28,304,585 total reads, 13,669,977 uniquely mapped
Antibodies	CTCF: 4uL per IP, Merk 07-729
Peak calling parameters	We used the ChIP-AP pipeline v5.4 for fully integrated ChIP-seq QC and peak calling, using the input chromatin samples as controls. Peaks called were calculated in ChIP-AP by default using MACS2, GEM, HOMER and Genrich. The ChIP-AP command was:<br>python ~/bin/chip-ap/chipap_installation/chipap_scripts/chipap.py --mode paired \\<br>--chipR1 ${RAW}/Mono1_CTCF_1.fq.gz ${RAW}/Mono2_CTCF_1.fq.gz ${RAW}/Mono3_CTCF_1.fq.gz \\<br>--chipR2 ${RAW}/Mono1_CTCF_2.fq.gz ${RAW}/Mono2_CTCF_2.fq.gz ${RAW}/Mono3_CTCF_2.fq.gz \\<br>--ctrlR1 ${RAW}/Mono1_input_1.fq.gz ${RAW}/Mono2_input_1.fq.gz ${RAW}/Mono3_input_1.fq.gz \\<br>--ctrlR2 ${RAW}/Mono1_input_2.fq.gz ${RAW}/Mono2_input_2.fq.gz ${RAW}/Mono3_input_2.fq.gz \\<br>--genome ~/bin/chip-ap/chipap_installation/chipap_scripts/genomes \\<br>--output ./chip-ap \\<br>--setname mono_CTCF \\<br>--homer_motif both \\<br>--run
Data quality	We ensured specificity to CTCF using homer motif enrichment analysis, within the ChIP-AP pipeline. There was strong enrichment for three CTCF motifs, with the top ranked motif overall being CTCF(Zf)/CD4+-CTCF-ChIP-Seq(Barski_et_al.)/Homer (40.87% of target regions versus 1.82% of background regions, p-value = 1e-13568).<br><br>No. peaks, under ChIP-AP default parameters:<br>MACS2: 26,834<br>GEM (GPS event peaks): 23,101<br>Homer: 28,456<br>Genrich: 30,349<br>Union (merged by ChIP-AP): 42,703

	We checked peaks by eye on a genome browser against publically available CTCF ChIP-seq in CD14 cells (available on the Remap database).
Software	We used the ChIP-AP pipeline v5.4 for fully integrated ChIP-seq QC and peak calling, using the input chromatin samples as controls.
